# Detection of genetic cardiac diseases by Ca^2+^ transient profiles using machine learning methods

**DOI:** 10.1038/s41598-018-27695-5

**Published:** 2018-06-19

**Authors:** Martti Juhola, Henry Joutsijoki, Kirsi Penttinen, Katriina Aalto-Setälä

**Affiliations:** 1 Faculty of Natural Sciences, University of Tampere, Tampere, Finland; 2 Faculty of Medicine and Life Sciences, University of Tampere, Tampere, Finland; 3 Heart Center, Tampere University Hospital, 33520 Tampere, Finland

## Abstract

Human induced pluripotent stem cell-derived cardiomyocytes (hiPSC-CMs) have revolutionized cardiovascular research. Abnormalities in Ca^2+^ transients have been evident in many cardiac disease models. We have shown earlier that, by exploiting computational machine learning methods, normal Ca^2+^ transients corresponding to healthy CMs can be distinguished from diseased CMs with abnormal transients. Here our aim was to study whether it is possible to separate different genetic cardiac diseases (CPVT, LQT, HCM) on the basis of Ca^2+^ transients using machine learning methods. Classification accuracies of up to 87% were obtained for these three diseases, indicating that Ca^2+^ transients are disease-specific. By including healthy controls in the classifications, the best classification accuracy obtained was still high: approximately 79%. In conclusion, we demonstrate as the proof of principle that the computational machine learning methodology appears to be a powerful means to accurately categorize iPSC-CMs and could provide effective methods for diagnostic purposes in the future.

## Introduction

## Basis of the current research

Induced pluripotent stem cell-derived^1^ cardiomyocytes (iPSC-CMs) have enabled the study of various genetic cardiac diseases such as catecholaminergic polymorphic ventricular tachycardia (CPVT)^2–9^, long QT syndrome (LQT)^10–13^ and hypertrophic cardiomyopathy (HCM)^14–16^, and all of these have revealed substantial abnormalities and diversity in Ca^2+^ cycling properties when compared with healthy controls. These Ca^2+^ abnormalities of different disease phenotypes have included irregularity, triggered action, and oscillations, but the variation of these Ca^2+^ transient profiles in different diseases remains unclear and unstudied. In CMs, Ca^2+^ cycling plays a central role in cardiac functionality by linking electrical activation and contraction, and characterization of Ca^2+^ cycling is vital in improving the study of disease pathology, prevention and treatment, but also, as shown in this study, in disease diagnostics.

Recently, we showed that normally beating iPSC-CMs can be efficiently distinguished from abnormally beating diseased CMs using signal analysis methods and different machine learning algorithms^17^. The categorization of Ca^2+^ transients in iPSC-CMs is a new analysis approach: the need for new analysis tools has grown after results of abnormal Ca^2+^ cycling have been obtained with several of the aforementioned iPSC-disease models. Computational analysis employing machine learning has offered new approaches in handling Ca^2+^ transient data recorded from different kinds of disease models^17,18^.

In the present study, we compared visually normal and abnormal Ca^2+^ transient signals and peak variables from three genetic cardiac diseases, including CPVT, an exercise-induced malignant arrhythmogenic disorder^4,9^; LQT type 1, an electric disorder of the heart that predisposes patients to arrhythmias and sudden cardiac death^13^; and HCM, a disorder that affects the structure of heart muscle tissue with increased risk of arrhythmias and progressive heart failure^16^. This comparison revealed that these diseases can be distinguished from each other based on our previously reported peak variable analysis^17^ computed from these Ca^2+^ signals.

In addition, controls (wild-type CMs, or WT), which included mainly normal Ca^2+^ transient signals recorded from healthy individuals, were compared with Ca^2+^ transient signals from three of the above-mentioned genetic cardiac diseases resulting furthermore high classification accuracies. Since there were only 13 abnormal control Ca^2+^ transient signals (9.8% of all control signals) –this is too small a number to be used as a disjoint group for machine learning methods – they were not used separately in tests. The classes were compared and classified in two main ways: first, normal signals of the controls compared separately to either normal or abnormal Ca^2+^ transient signals of the three diseases, and second, signals of the combined normal and abnormal signals of the controls compared to combined normal and abnormal signals of each of the three diseases, i.e. four classes in total.

## Methods

This study was approved by the Ethics Committee of Pirkanmaa Hospital District in establishing, culturing, and differentiating hiPSC lines (R08070). The study protocol was explained to all subjects (fibroblast donors), and they all gave their informed consents. All experimental methods were carried out in accordance with approved guidelines. Patient-specific iPSC lines were established and characterized as described earlier^9,13,16^. Studied cell lines included six CPVT lines generated from CPVT patients carrying cardiac ryanodine receptor (RyR2) mutations: four HCM cell lines generated from HCM patients carrying either α-tropomyosin (TPM1) or myosin-binding protein C (MYBPC3) mutations, two LQT type 1 cell lines generated from patients carrying potassium voltage-gated channel subfamily Q member 1 (KCNQ1) mutations, and one cell line generated from a healthy control individual. All the cell lines and their mutations are shown in Supplementary Table 1. The iPSCs were differentiated into spontaneously beating CMs using the END2 differentiation method^19^ and dissociated to single-cell level for Ca^2+^ imaging studies, which were conducted in spontaneously beating Fura-2 AM (Invitrogen, Molecular Probes) loaded CMs as described earlier^2^. Briefly, CMs were perfused with 37 °C perfusate consisting of (in mM) 137 NaCl, 5 KCl, 0.44 KH2PO4, 20 HEPES, 4.2 NaHCO3, 5 D-glucose, 2 CaCl2, 1.2 MgCl2, and 1 Na-pyruvate (the pH was adjusted to 7.4 with NaOH). Ca^2+^ measurements were conducted on an inverted IX70 microscope with a UApo/340 x20 air objective (Olympus Corporation, Hamburg, Germany) and images taken with an ANDOR iXon 885 CCD camera (Andor Technology, Belfast, Northern Ireland) and synchronized with a Polychrome V light source by a real time DSP control unit and TILLvisION or Live Acquisition (TILL Photonics, Munich, Germany) softwares. Ca^2+^ signals were acquired as the ratio of the emissions at 340/380 nm wavelengths, and background noise was subtracted before further processing. Each Ca^2+^ signal corresponded to a recording from one cell.

### Data computed from signals measured

**Figure 1:**
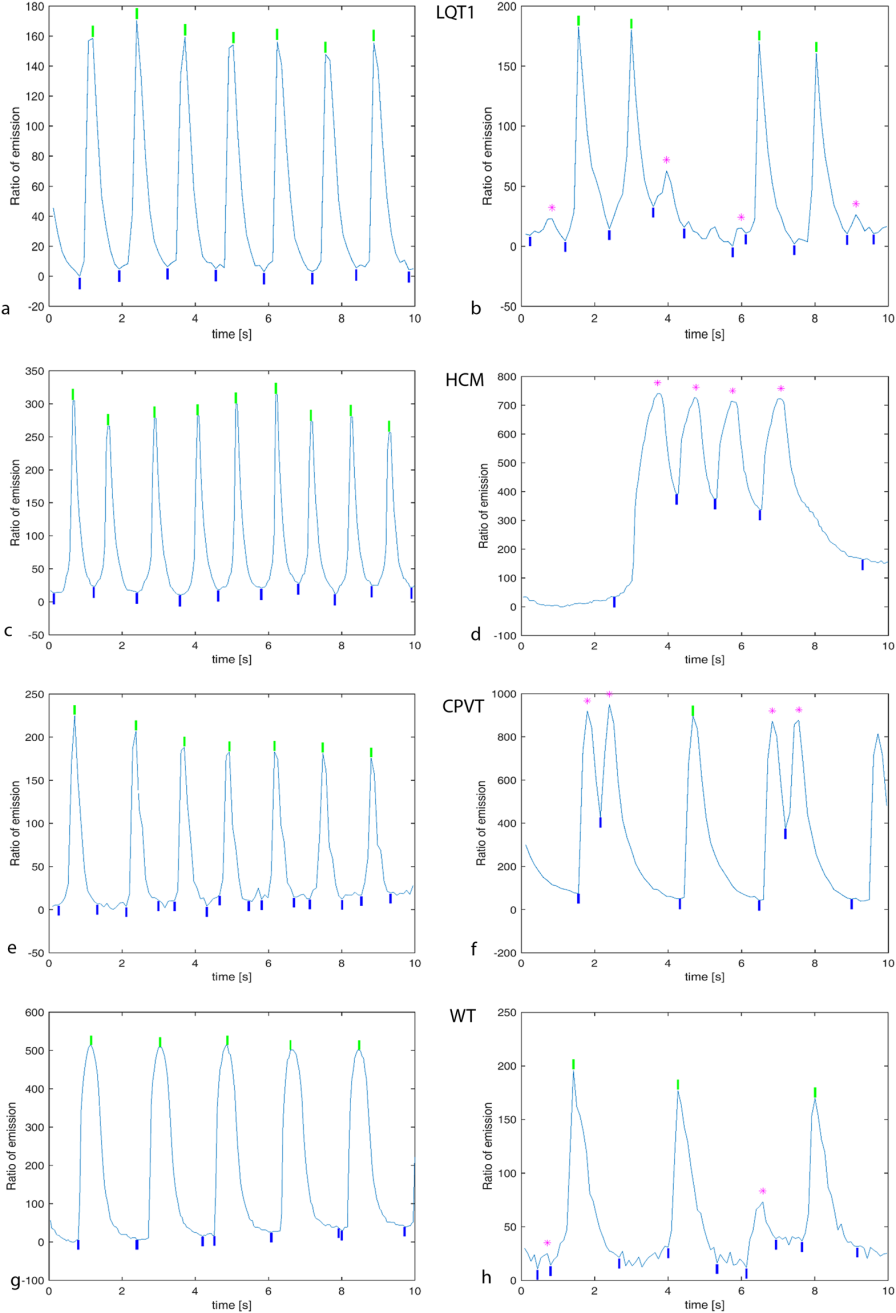
Example signals of Ca^2+^ transients. (**a)** 10 s from a normal LQT1 signal; entire peaks were detected as normal by the peak detection algorithm. (**b)** From an abnormal LQT1 signal; four peaks were detected as normal, but the four small peaks marked with purple asterisks at the tops as abnormal. (**c)** From a normal HCM signal; peaks detected as normal. (**d)** From an abnormal HCM signal; four peaks marked with purple detected as abnormal. (**e)** From a normal CPVT signal; peaks detected as normal. (**f)** From an abnormal CPVT; four peaks detected as abnormal marked with purple. (**g**) From a normal control signal. (**h**) From an abnormal control signal abnormal peaks marked with purple. The maxima (tops) of normal peaks are marked with green vertical bars; the beginnings and endings of all peaks are marked with blue vertical bars.

In order to compute relevant data variables, the peaks from Ca^2+^ transient signals were detected, after which peak variables were computed from detected peaks. Ca^2+^ transient signals were categorized using our recognition algorithm^17^, which classified every peak into either normal or abnormal type (Fig. 1a–h). If even a single peak of a signal was classified as abnormal, the entire signal was seen as abnormal (Fig. 1b,d,f,h). However, instead of the fully automatic classification of entire Ca^2+^ transient signals as normal or abnormal, a human expert was also used to determine their classes to obtain certainty of correctness of classification. In our previous research^17^, we developed an automatic method whose results agreed with the expert’s classification with an accuracy of around 90%. In addition, the human expert checked visually that the beginning, top and end of each peak were properly detected in every signal. This was done simply to guarantee the high quality of the peak data to be used in the subsequent classification.

**Figure 2:**
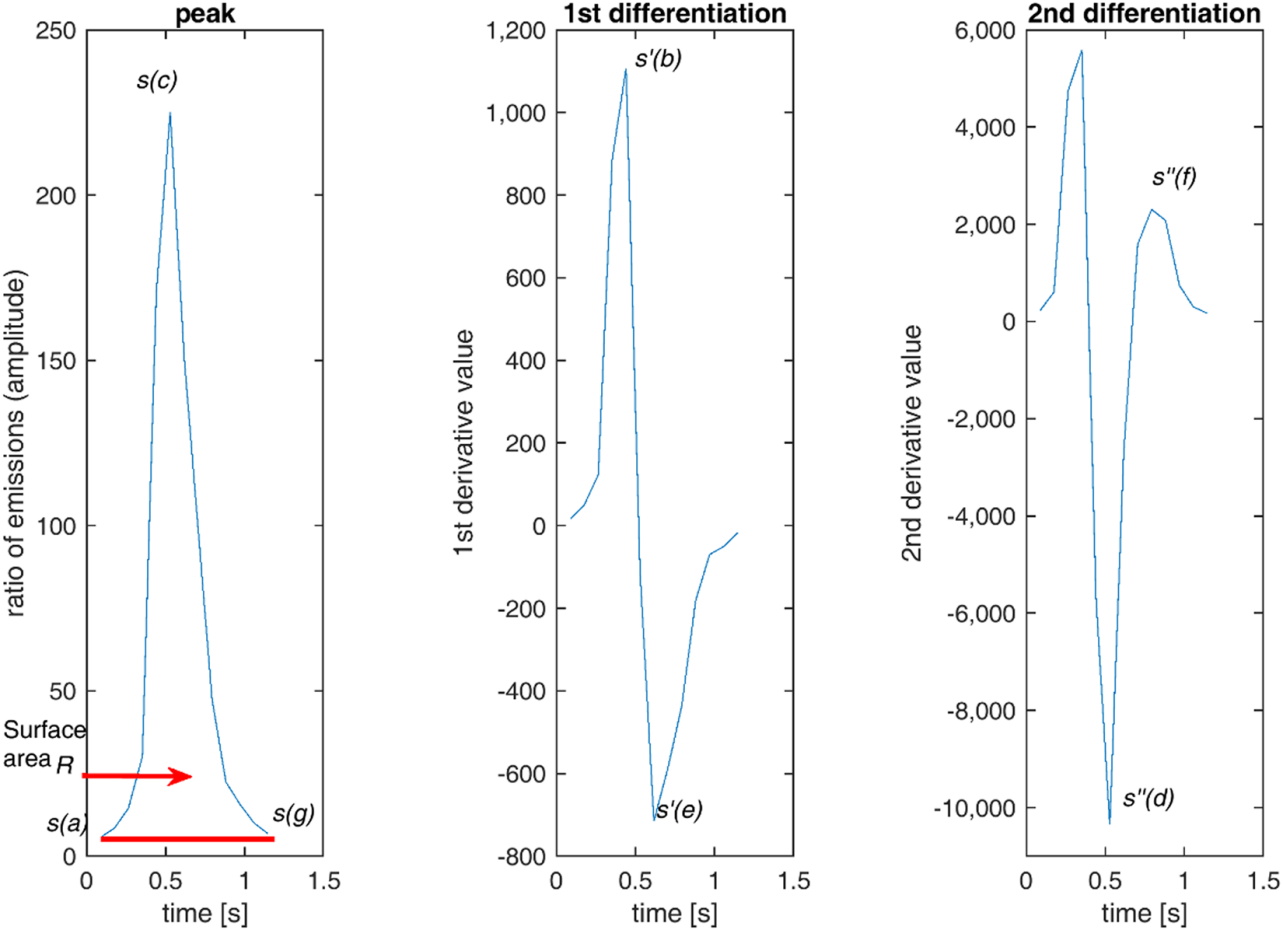
The first peak classified as normal from Fig. 1e. In the left panel, the peak curve is *s*. *Variables*: Left amplitude *A*_*l*_ is the difference between curve locations of peak beginning *s*(*a*) and maximum at location *s*(*c*). Right amplitude *A*_*r*_ is the difference between end *s*(*g*) and *s*(*c*). Duration *D*_*l*_ of the peak left side is time difference from *a* to *c* along the horizontal axis. Duration *D*_*r*_ of the peak right side is time difference from *c* to *g*. Peak-to-peak time difference Δ is normally computed from the current peak maximum to that of the preceding peak. Exceptionally for the first peak of the signal, it is time difference from the first peak maximum to the signal beginning. The surface area *R* is formed by curve *s* and line from *s*(*a*) to *s*(*g*). In the middle panel, the first derivative *s*′ of the peak contains the maximum at location *s*′(*b*) and minimum at *s*′(*e*). In the rightmost panel, the second derivative *s*′′ is obtained by extracting the right segment from *c* to *g* based on the peak of the left panel and its minimum is at location *s*′′(*d*) and maximum at *s*′′(*f*). (The horizontal axis is scaled in seconds to express time clearly, but computation was performed according to the formulas given in the section entitled “method*s*′′. The symbols *a*, *b*, *c*, *d*, *e*, *f* and *g* are index values of signals. The directly time-related variables are computed by dividing them with the sampling frequency *F*.).

Ten peak variables, as presented in Fig. 2, were defined as follows: (1) amplitude of peak left side *A*_*l*_, (2) amplitude of peak right side *A*_*r*_, (3) duration of peak left side *D*_*l*_, (4) duration of peak right side *D*_*r*_, (5) maximum of the first derivative *s*′ on the left side of a peak, (6) absolute minimum *s*′ of the first derivative on the right side of a peak, (7) maximum of the second derivative *s*′′ on the right side of a peak, (8) absolute minimum *s*′′ of the second derivative on the right side of a peak, (9) area of a peak *R*, and (10) time difference Δ from peak to peak. Amplitudes (heights) of peak left and right sides (Fig. 2, left panel) were computed based on the values of signal *s* according to

1 $${A}_{l}=s(c)-s(a)\,{\rm{a}}{\rm{n}}{\rm{d}}\,{A}_{r}=s(c)-s(g),$$

durations of the left and right sides of a peak, where *F* is the sampling frequency, and

2 $${D}_{l}=\frac{c-a}{F}\,{\rm{a}}{\rm{n}}{\rm{d}}\,{D}_{r}=\frac{g-c}{F},$$

the maximum of the first derivative *s*′(*b*) on the left side of a peak, the absolute minimum *s*′(*e*) of the first derivative on the right side of a peak in Fig. 2 (middle panel), and the time difference from the current peak to the preceding one Δ, which was computed from the peak maximum (top) to that of the preceding peak. For the first peak of a signal, the time difference was calculated from the maximum (top) of the first peak to the signal beginning. Additionally, the maximum *s*′′(*f*) of the second derivative on the right side, absolute minimum *s*′′(*d*) of the second derivative on the right side (Fig. 2, right panel) and surface area *R* of the entire peak in Fig. 2 (left panel) computed with formula

3 $$R=\sum _{i=2}^{{n}_{j}}\frac{s(i)+s(i-1)}{2F}-\frac{(g-a)s(g)}{2F}$$

in which *n*_*j*_ is the number of samples of the *j*th peak in the signal evaluated. The trapezoidal rule was employed here in order to approximate the surface area defined by the definite integral determined by the nonlinear signal *s*(*t*).

### Data analysis actions

Data analysis consisted of two separate parts based on the peak variable values computed: preliminary analysis and main analysis. The preliminary analysis consisted of the computation of statistical results for which one-way variance analysis was executed to denote the chance of separating three (three cardiac diseases) or four (three diseases and controls) different data classes from each other. In addition, our Scatter method^20^ was applied to the data in order to evaluate how efficient the ten peak variables were for the classification task. The Scatter method analyzes the separating power of data sets in which the class labels corresponding to three diseases and controls are known. The Scatter method evaluated how extensively the cases of the labeled classes were located in separate parts of the variable space formed by the ten variables: the more separate the classes, the higher the separating power value from [0, 1] achieved.

The main analysis included classification of the three diseases studies, i.e., separation from each other with different classification methods based on machine learning. The class of controls was added to be the fourth class of classification. Machine learning means creating computational models using the current data, and, in this study, these models were intended for use in predicting a cardiac disease class or control class (WT) for novel cases provided from among the four classes given here. In the computational sense, such computational models can be conceived as generalizations for either these three diseases (LQT1, HCM or CPVT) or four classes, including also controls based on the current data. Ca^2+^ transient signals data was classified according to leave-one-out principle^21^, in which a computational model was constructed using a machine learning method with *n* − 1 training signals (their peak variables) from the whole data set of *n* Ca^2+^ transient signals. After this, the model constructed was tested using the single signal. This procedure was repeated *n* times by taking each signal once to be the test signal and the rest, *n* − 1, as the training set for a model.

### Code availability

The data used in the current research and the programs implemented to produce the results presented are available on request via the link www.biomeditech.fi/calciumdiagnostics.

## Results

Figure 1 presents examples of Ca^2+^ peak detection from all the studied diseases: LQT type 1 in Fig. 1a,b, HCM in Fig. 1c,d, and CPVT in Fig. 1e,f, with both abnormal and normal Ca^2+^ cycling. Figure 1g shows a normal control (WT) signal and 1 h an abnormal control (WT) signal. Figure 2 shows how peak variables were formed. The shapes and sizes of Ca^2+^ transient peaks occasionally varied greatly in different signal recordings from the same cell line and even in peaks within individual recordings, particularly for abnormal signals. Normal Ca^2+^ transients included successive harmonious peaks in Fig. 1a,c,e,g. The abnormal signal in Fig. 1b contained smaller peaks than those assessed to be normal, and the abnormal signals in Fig. 1d,f contained peaks that were asymmetric in their left- and right-side amplitude heights or peak shapes that were deformed compared with more the regular shapes encountered in normal Ca^2+^ transient signals. The abnormal signal in Fig. 1h consisted of two small peaks. Very small peaks with low amplitudes (less than 2% compared with the maximum peak amplitude inside each signal) were considered noise and excluded.

### Data analysis of computed variable values

After having recognized peaks and computed their peak variables, we first studied whether the properties of Ca^2+^ transients from three different diseases differed from each other. Our data set from Supplementary Table 1 included 90 signals from LQT1, 71 signals from HCM, and 233 signals from CPVT CMs, a total of 394 signals. Each Ca^2+^ signal corresponded to a recording from one cell. After this, the influence of adding the 133 control (WT) signals to the data set and results was studied.

Approximate sampling frequencies were 8.3 Hz for 146 signals, 11 Hz for 133 signals and 23 Hz for 248 signals. Since signals were measured during a couple of years, the measurement software were updated twice which improved (increased) the sampling frequency. LQT1 signals were of 8.3 Hz, HCM signals of 23 Hz, and CPVT mostly of 11 Hz and 23 Hz, and WT signals mostly of 23 Hz.

First, means and standard deviations were computed for the Ca^2+^ transient signals of the three diseases and controls, and all Ca^2+^ transients (normal and abnormal) of each type of diseased CMs were combined in the analysis. Thus, all 527 Ca^2+^ transient signals were considered in the whole of four classes: three diseases and controls. Table 1 represents the means and standard deviations of all ten variables for every disease class and controls, which clearly shows that the means vary between the four classes.

**Table 1:** Means and standard deviations for the ten peak variables of Ca^2+^ transient signals.

Disease or control	Variables									
	*A*_*l*_	*A*_*r*_	*D*_*l*_ [s]	*D*_*r*_ [s]	max *s*′	|min s’|	max *s*′′	|min *s*′′|	*R*	Δ [s]
LQT1	170 ± 79	172 ± 80	0.33 ± 0.18	0.68 ± 0.40	817 ± 472	508 ± 259	1,615 ± 1,324	1,208 ± 1,432	58 ± 42	1.17 ± 0.92
HCM	191 ± 89	193 ± 91	0.23 ± 0.12	0.43 ± 0.24	1,990 ± 920	1,052 ± 469	6,420 ± 3,382	3,235 ± 2,905	43 ± 36	0.72 ± 0.47
CPVT	229 ± 176	232 ± 176	0.34 ± 0.20	0.63 ± 0.43	1,349 ± 1,064	812 ± 541	2,895 ± 2,535	2,106 ± 2,709	85 ± 103	1.13 ± 0.94
WT	320 ± 189	323 ± 190	0.46 ± 0.21	0.79 ± 0.36	2,189 ± 1,203	1,161 ± 667	5,122 ± 3,432	4,048 ± 4,151	130 ± 104	1.48 ± 0.75

Results for 1635 peaks of LQT1, 1344 peaks of HCM, 2311 peaks of CPVT diseases and 1216 peaks of controls (WT): amplitude of peak left side *A*_*l*_, amplitude of peak right side *A*_*r*_, duration of peak left side *D*_*l*_, duration of peak right side *D*_*r*_, maximum of the first derivative *s*′ on the left side of a peak, absolute minimum *s*′ of the first derivative on the right side of a peak, maximum of the second derivative *s*′′ on the right side of a peak, absolute minimum *s*′′ of the second derivative of the right side of a peak, area *R* of a peak, and time difference Δ from peak to peak.

Second, one-way variance analysis was computed for all six pairs of the three diseases and controls for every variable of the data of the normal and abnormal signals together in Supplementary Table 2: {LQT1 vs. HCM, LQT1 vs. CPVT, LQT1 vs. WT, HCM vs. CPVT, HCM vs. WT, CPVT vs. WT}. All pairs for all peak variables (six pairs times ten variables), excluding two variables for the pair of LQT1 vs. CPVT, differed significantly (*p* < 0.001). See also Supplementary Figure 1. These two exceptional peak variables were duration of the left peak sides and peak-to-peak time difference. However, the peak variable representing duration of the left right peak side differed significantly (*p* < 0.02) in the LQT1 vs. CPVT comparison. Thus, 58 of the 60 comparisons were statistically highly significant, and an additional one of the 60 comparisons was statistically significant. This predicts good opportunities for separating both the three diseases and controls from each other.

Third, the Scatter method^20^ enabled the evaluation of a single separating power value for the whole data set, for all classes and for all variables. The values computed are shown in Supplementary Table 3. The single separation power value of the whole data set and the four classes is (relatively) high compared with the real data sets for which the corresponding values have been computed so far. For instance, previously^20^, the mean separation power of 24 classes in six data sets was 0.29. When evaluating the separation power of peak variables, three of them – i.e., durations of the left and right peak sides and peak-to-peak time difference – were all associated with time and obtained high separation power values. These outcomes predict a highly promising start for the separation of the classes LQT1, HCM, CPVT and WT.

### Classification of Ca^2+^ transient signals

At first, all data of each peak variable were standardized which is necessary for some classification methods, particularly, those of nearest neighbor searching. There were two main classification arrangements: the one for three disease classes and the other for both three diseases and controls, i.e., a total of four classes. In addition, normal and abnormal transient signals were taken into account as follows.

Classification alternatives

1. Three diseases:
  (1.1) Classification of only normal signals in the three diseases
  (1.2) Classification of only abnormal signals in the three diseases
  (1.3) Classification of both normal and abnormal signals together in the three diseases
2. Three diseases and controls (four classes):
  (2.1) Classification of only normal signals in the three diseases and controls
  (2.2) Classification of both normal and abnormal signals together in the three diseases and controls in each of these four classes

The purpose here was to study how three diseases differ from each other in respect of the data and how they differ from controls. It was essential to study whether they differed sufficiently to enable their computational modeling through machine learning.

At first, tests were performed separately for the (1.1) normal and (1.2) abnormal signals. After classification using several classification methods^21,22^, the true positive rates or sensitivity (ratio of the correctly classified signals of a disease and their total) and accuracy (ratio of correctly classified signals for all diseases and the number of all signals) were computed. Next, classifications were executed by combining (1.3) the normal and abnormal signals for each disease class. This classification is important because it implements the possible future diagnostic practice of prior Ca^2+^ signal analysis not being required but instead all cells being analyzed and pooled. An exact presentation of the machine learning classification methods used is given in the Supplementary Note. Secondly, four classes of the three diseases and controls were classified. Either the diseases classes consisted of (2.1) only normal signals or they consisted of (2.2) both normal and abnormal signals. Abnormal signals were not tested separately because the class of the controls only contained 13 signals, which is too few for most machine learning methods.

Supplementary Table 4 shows the classification results yielded by several classifiers based on various classification methods for the (1.1) normal signals of the three diseases. The results in Supplementary Table 4 for the normal signals of the three diseases show that random forest was the best of classifiers, with an accuracy of 86.0%, and LS-SVM with a radial basis function kernel (RBF) the second best, with an accuracy of 84.4%. Correspondingly, in Supplementary Table 5 for the (1.2) abnormal signals of three diseases, random forest classifier provided the best accuracy of 86.0%, and the classifiers of LS-SVM RBF and *k*NN with Mahalonobis metric the next best accuracy of 85.1%. By applying all the same classifiers as in Supplementary Tables 4 and 5, the tests (1.3) were computed, but only the highest-accuracy results were shown in Table 2. The best accuracy, 87.6%, was achieved with random forest.

**Table 2:** Both normal and abnormal signals of three diseases.

Classification method	TPR of LQT1	TPR of HCM	TPR of CPVT	Accuracy
*k*NN, cityblock metric, equal weighting, *k* = 1	86.7	87.3	80.7	83.2
*k*NN, cityblock metric, inverse weighting, *k* = 5	94.4	84.5	79.0	83.5
*k*NN, cityblock metric, squared inverse weighting, *k* = 5	91.1	85.9	81.5	84.5
Random forests, 35 trees	88.9	84.5	88.0	**87**.**6**
LS-SVM cubic kernel, parameter *C* = 2^−5^	87.8	78.9	85.4	84.8
LS-SVM RBF kernel, parameters *C* = 2, sigma = 2	90.0	78.9	88.4	**87**.**1**

True positive rates (TPR, %) for LQT1, HCM and CPVT diseases, with 90, 71 and 233 signals respectively, and accuracy (%) of all signals (*k*NN is *k* nearest neighbor searching method and LS-SVM RBF least square support vector machine with a radial basis function kernel). The best accuracies are bolded.

Ultimately, Supplementary Table 6 presents the highest-accuracy results for (2.1) using the normal signals of diseased and control CMs. Three classifiers of *k*NN with Mahalonobis metric produced the best classification accuracy of 76.3%. Table 3 presents the highest accuracies for (2.2) using both the normal and abnormal signals of the diseased CMs and the normal signals of the control CMs. The random forests gave the best classification accuracy: 78.6%. These high classification accuracies indicate that the three diseases and controls can be separated efficiently from each other on the basis of the present peak variable data computed from CMs.

**Table 3:** Normal and abnormal signals together of three diseases and controls.

Classification method	TPR of LQT1	TPR of HCM	TPR of CPVT	TPR of WT	Accuracy
*k*NN, cityblock metric, equal weighting, *k* = 5	93.3	76.1	70.4	68.4	74.6
*k*NN, cityblock metric, inverse weighting, *k* = 5	93.3	74.6	71.7	68.4	75.0
*k*NN, cityblock metric, squared inverse weighting, *k* = 5	91.1	76.1	71.7	69.2	75.0
*k*NN, Mahalanobis metric, equal weighting, *k* = 1	87.8	80.3	71.7	69.2	75.0
*k*NN, Mahalanobis metric, inverse weighting, *k* = 1	87.8	80.3	71.7	69.2	75.0
*k*NN, Mahalanobis metric, squared inverse weighting, *k* = 11	94.4	78.9	71.2	66.9	75.1
Random forests, 54 trees	88.9	81.7	76.8	72.9	**78**.**6**
LS-SVM RBF kernel, parameters *C* = 2^4^, sigma = 2	85.6	71.8	70.8	78.2	**75**.**3**

True positive rates (TPR, %) of LQT1, HCM, CPVT diseases and controls (WT) with 90, 71, 233 and 133 signals respectively and accuracy (%) of all signals (*k*NN is *k* nearest-neighbor searching method and LS-SVM least square support vector machine). The best accuracy is bolded.

In classification, a random guess is seen as a baseline result when there is a model that would not hypothetically have any other information about data than there are four different classes. Then a random guess would be equally 25% for each class as well as for accuracy in general. If the class distribution of four classes were known, there would be slightly more information about data in the sense of probability or information theory. Now the hypothetical model would classify according to the majority class being the most frequent in the data predicting that all test cases would be from the majority class. This would give the accuracy of 44.2% because of 233 signals of the largest CPVT class among all 527 signals. These two accuracy values are now theoretical baselines. Accuracies that are considerably higher than the preceding two would then be results from models that are able to classify clearly more successfully than randomly or by a majority class. In practice, depending on data sets, it may vary even tens of per cents which are reasonable accuracies. For example, in the recent research^20^ with six different medical data sets by using nearest neighbor searching method we obtained the accuracies of 61% for a data set of two classes (low accuracy because of only two classes), 94% for a data set of three classes (excellent accuracy), 73% for two classes, 76% for two classes (good accuracies), 78% for five classes and 73% for ten classes (very good accuracies because of more classes than in the preceding “easier” data sets). The different results reflect the complexities of the classification tasks depending on the properties of a data set.

## Discussion

The iPSC modelling of human cardiac disorders enables the study of disease pathophysiology and the development of therapies, but it can also, as shown in this study, offer a tool for disease diagnostics. Some computational machine learning methods, particularly random forests and the least square support vector machine with an RBF kernel, including the computation of Ca^2+^ transient peak variable values, were shown to be a powerful tool to accurately separate the Ca^2+^ transient signals of the three diseases – including LQT1, HCM, and CPVT – from each other and from control WT iPSC-CMs with high classification accuracies (79–88%). This strongly indicates the possibility of discriminating between genetic cardiac diseases using Ca^2+^ transient profiles recorded from iPSC-CMs with signal analysis and machine learning classification methods. By combining the normal and abnormal signals for each disease class and showing the high classification accuracy, it was demonstrated as a proof of principle that, in the future, prior Ca^2+^ signal analysis will not be required in diagnostic practice, but all cells can be pooled and analyzed.

Since the differences between the average peak variable values and the value distributions computed from the three cardiac diseases and control data were considerable, this provided a good opportunity for classification. Our results indicated that, subject to high classification accuracies, the peak data computed from the Ca^2+^ transient signals of iPSC-CMs enable the reliable classification of such signals into these four classes. This novel finding is also an excellent starting point for additional utilization of machine learning methods for Ca^2+^ transient signals from other inherited cardiac diseases in the future. As a study limitation, it should be stated that for generation of future clinical diagnostic tool more data from control hiPSC-CMs from various individuals should be compared and included for optimization of the software and classifications. This would also require standardizations of the Ca^2+^ transient signals and machine learning methods.

Machine learning is one of the most important areas of artificial intelligence. Its importance comes from the essential property of enabling learning from data for the purpose of building computational models, particularly for use in classification. Several different machine learning methods have been developed since the 1960s: from nearest neighbor searching^23^ as one of the earliest to the “newest” support vector machines in the 1990s^24^ and random forest in the early 2000s^25^. In fact, these three machine learning algorithm types yielded the best results for the current data as presented in Tables 2 and 3. Frequently, we have drawn similar conclusions from our other recent machine learning studies, for example, for eye movement signal data^26^ and socio-economic crime data^27^. Although these machine learning methods were developed several years or even decades ago, they have in recent years become highly useful with the innumerable data sources that have arisen and along with global digitalization and the development of various measurement and data collecting technologies. No doubt, there are now key computational algorithms to solve in machine learning: among other things, classification problems for use in almost any areas in which data can be collected efficiently. Eye movements could be used for verification and identification of subjects, for example, as users of computers^26^ (to replace passwords); country crime data can be used to study crime rates and types^27^ for comparison with different countries on the basis of databases from the United Nations and national statistical organizations; or freshwater benthic macroinvertebrates^28^ (small aquatic animals) could be classified for water quality research in inland waterways. One of the most interesting fields to utilize machine learning is medicine. For instance, personalized medicine, medical imaging and diagnostics of diseases will, obviously, derive great advantages from machine learning, as demonstrated in this current study.

Genetic testing of inherited cardiac diseases has increased enormously over the past 20 years. The number of genes and gene variations involved in different diseases has increased, but also the prevalence of findings of unknown significance has multiplied^29^. Additionally, penetrance of potential disease-causing variations is often incomplete or manifests only later in life, thus complicating and delaying the diagnosis of inherited cardiac diseases^30^. The clinical phenotype could also be affected by other factors, e.g. the background genome of the individual and many environmental or lifestyle factors, thus complicating diagnosis and delaying potentially preventive medication. Often clinical findings guide diagnostic tests towards a certain genetic disease, e.g. the presence of a prolonged QT interval or myocardial hypertrophy. However, sudden cardiac death at a relatively young age is still often the only clinical observation in the family, and genetic tests employing current genetic analysis methods can only suggest potentially disease-causing variation or variations of unknown significance. In these cases, iPSC-CMs could provide an additional tool in diagnostics. In practice, this would mean that iPSC-CMs would be produced from individuals with family background of sudden cardiac death and/or with unspecific clinical symptoms. The calcium signals of these cells would be measured and used for detection of potential genetic cardiac diseases by Ca^2+^ transient profiles using machine learning methods. Further clinical investigations or treatment options could be at least partially guided by the results obtained by the machine learning algorithm. Currently, the protocol for obtaining iPSC-CMs is fairly long and does not apply to most patients, but with better and more advanced methods in the future, e.g. direct differentiation of blood cells into CMs^31^, they could provide a realistic additional tool for diagnosing genetic cardiac disease. Computational machine learning methods could become an automated, high-throughput software tool to assist diagnostics in the future.

Whole-cell Ca^2+^ transients have been extensively characterized from iPSC-CMs, and this has demonstrated the presence of a functional excitation-contraction coupling that resembles the native myocardium^32,33^. Several previous studies have shown Ca^2+^ cycling abnormalities and irregularities in CPVT^2–9^, HCM^14–16^, and LQT1^13^ -specific iPSC-CMs. In addition, healthy control CMs have shown mostly normal and constant Ca^2+^ cycling. In spite of this, Ca^2+^ cycling kinetics in iPSC-CMs have been claimed to be relatively slow and sometimes characterized by a U-shape Ca^2+^ waveform, suggesting that iPSC-CMs could have an immature CICR mechanism^34^. Some have also reported poorly developed SR and an absence of T-tubules, which can also affect Ca^2+^ handling properties of these cells^35,36^. This could indicate that iPSC-CMs may not represent the disease phenotype accurately, but, as we demonstrate here, with specific peak variable computations, these signals can be separated into different disease-specific iPSC-CMs and healthy iPSC-CMs. The classifications using abnormal Ca^2+^ transient signals resulted in only a slightly higher classification accuracy than if visually normal signals of the diseased CMs were analyzed alone. Thus, it can be stated that diseased and control iPSC-CMs differ slightly more in abnormal Ca^2+^ transient signals than in normal signals, which is an expected observation, since abnormal signals are thought to represent the phenotype of the diseased CMs. However, to our knowledge, this is the first time that it has been shown that also visually normal Ca^2+^ transient signals in diseased CMs and their peak variables differ significantly from healthy controls, also when different disease phenotypes are compared with each other. Additionally, if both normal and abnormal Ca^2+^ transient signals in each disease were pooled, the classification accuracy of each disease was equally good as it was if only abnormal signals were analyzed. This underlines the importance of peak variable analysis: although especially normal signals may seem visually similar in different diseases and also in controls, there is still a need for rigorous analysis.

As a study limitation, it must be noted that Ca^2+^ indicators can affect to the physiology of the cells. The addition of a chemical indicator, which attaches to molecules inside the cell, is likely to interfere with the functioning of the cell, which must be always kept in mind when interpreting the results^37^. Currently available and widely-used Ca^2+^ indicators, such as Fura-2 and Fluo-4, are toxic, and UV-light, which is needed in some recording, is also harmful for the cells^38,39^. Limitation of these indicators is also inability to obtain long-term recordings. (Shinnawi *et al*.^39^) It has been also been criticized that these widely-used indicators have a relatively high affinity for Ca^2+^, which can artificially prolong the Ca^2+^ transient and confound interpretation^40^. Limitation with ratiometric indicators such as Fura-2 is the low temporal resolution resulting from the requirement to monitor at two excitation or emission wavelengths and relatively small dynamic ranges^41^. The most ideal Ca^2+^ indicator molecule would combine the option of ratiometry for amplitude quantification with low Ca^2+^ affinity^40^. Fusion genes based on green fluorescent protein (GFP) have been developed to circumvent issues with chemical indicators, but might affect the folding and functioning of the proteins in the cell, and still needs the UV excitation^37^. Genetically encoded indicators offer photo stability, excellent signal-to-noise ratio and minimal cellular-toxicity^39^ but their well-known limitation is their slow response time because of the slow on and off kinetics of calcium binding^40^. It can be concluded that the ideal and optimal calcium indicator is still missing but so far, the iPSC disease modeling studies using well-known chemical indicators have been able to recapitulate well clinical phenotypes of the diseased patients.

Cardiomyocyte functionality can be assessed on different levels, starting from the analysis of single cells all the way to studying the entire organ. Earlier numerous studies have demonstrated that single iPSC-CMs display physiologically relevant characteristics and patient-derived iPSC-CMs recapitulate aspects of patient cardiac pathology/phenotype *in vitro*^2–4,6–16^ as well as clinically-relevant drug responsiveness^9^. In this study we chose to analyze single cardiomyocytes and their Ca^2+^ transients in diseased and healthy state and these machine learning algorithms presented here are currently based on analyzing single cardiomyocytes. The suitability of machine learning in predicting disease states in larger cell aggregates or tissue samples is to be explored.

In the future, this machine learning classification method approach could be utilized in diagnosing genetic cardiac disease and evaluating arrhythmia risks in individuals. The method could possibly be optimized to study patient-specific drug therapy, and in improving drug and treatment efficiency. This method could help to better diagnose genetic arrhythmias and to provide more accurate information about the disease, as well as uncover the differences among people that could influence their response to therapies.

## Electronic supplementary material

Supplementary Information

## References

[1] Takahashi K (2007). Induction of pluripotent stem cells from adult human fibroblasts by defined factors. Cell.

[2] Fatima A (2011). *In vitro* Modeling of Ryanodine Receptor 2 Dysfunction Using Human Induced Pluripotent Stem Cells. Cell Physiol. Biochem..

[3] Jung CB (2012). Dantrolene rescues arrhythmogenic RYR2 defect in a patient-specific stem cell model of catecholaminergic polymorphic ventricular tachycardia. EMBO Mol. Med..

[4] Kujala, K. *et al*. Cell model of catecholaminercig polymorphic ventricular tachycardia reveals early and delayed after depolarizations. *PlosONE* **7****(**9), 10.1371/journal.pone.0044660 (2012).

[5] Novak A (2015). Functional abnormalities in iPSC-derived cardiomyocytes generated from CPVT1 and CPVT2 patients carrying ryanodine or calsequestrin mutations. J. Cell. Mol. Med..

[6] Itzhaki I (2012). Modeling of catecholaminergic polymorphic ventricular tachycardia with patient-specific human-induced pluripotent stem cells. J. Am. Coll. Cardiol..

[7] Zhang XH (2013). Ca2+ signaling in human induced pluripotent stem cell-derived cardiomyocytes (iPS-CM) from normal and catecholaminergic polymorphic ventricular tachycardia (CPVT)-afflicted subjects. Cell Calcium.

[8] Di Pasquale E (2013). CaMKII inhibition rectifies arrhythmic phenotype in a patient-specific model of catecholaminergic polymorphic ventricular tachycardia. *Cell*. Death Dis..

[9] Penttinen, K. *et al*. Antiarrhythmic effects of Dantrolene in patients with catecholaminergic polymorphic ventricular tachycardia and replication of the responses using iPSC models. *PlosONE* **10**(7), 10.1371/journal.pone.0125366 (2015). 10.1371/journal.pone.0125366 PMC4425399 25955245

[10] Moretti A (2010). Patient-specific induced pluripotent stem-cell models for long-QT syndrome. N. Engl. J. Med..

[11] Matsa E (2011). Drug evaluation in cardiomyocytes derived from human induced pluripotent stem cells carrying a long QT syndrome type 2 mutation. Eur. Heart J..

[12] Lahti AL (2012). Model for long QT syndrome type 2 using human iPS cells demonstrates arrhythmogenic characteristics in cell culture. Dis. Model. Mech..

[13] Kiviaho AL (2015). Distinct electrophysiological and mechanical beating phenotypes of long QT syndrome type 1-specific cardiomyocytes carrying different mutations. IJC Heart & Vasculature.

[14] Han L (2014). Study familial hypertrophic cardiomyopathy using patient-specific induced pluripotent stem cells. Cardiovasc. Res..

[15] Lan F (2013). Abnormal calcium handling properties underlie familial hypertrophic cardiomyopathy pathology in patient-specific induced pluripotent stem cells. Cell. Stem Cell..

[16] Ojala, M. *et al*. Mutation-specific phenotypes in hiPSC-derived cardiomyocytes carrying either myosin-binding protein C or α-Tropomyosin Mutation for Hypertrophic Cardiomyopathy. *Stem Cells Int*., https://www.hindawi.com/journals/sci/2016/1684792/ (2016). 10.1155/2016/1684792 PMC4707351 27057166

[17] Juhola M (2015). Signal analysis and classification methods for the transient data of stem cell-derived cardiomyocytes. Comp. Biol. Med..

[18] Heylman C, Datta R, Sobrino A, George S, Gratton E (2015). Supervised machine learning for classification of the electrophysiological effects of chronotropic drugs on human induced pluripotent stem cell-derived cardiomyocytes. PlosONE.

[19] Mummery C (2003). Differentiation of human embryonic stem cells to cardiomyocytes: role of coculture with visceral endoderm-like cells. Circulation.

[20] Juhola M, Siermala M (2012). A scatter method for data and variable importance evaluation. Integr. Comp.-Aided Eng..

[21] Witten, I. H., Frank, E. & Hall, M. A. Data Mining, third ed., (Morgan Kaufmann, Burlington, MA, USA, 2011).

[22] Webb, A. Statistical Pattern Recognition, second ed., John Wiley & Sons, (Chichester, England, 2002).

[23] Cover TM, Hart PE (1967). Nearest neighbor pattern classification. IEEE Trans. Inf. Theory.

[24] Cortes C, Vapnik V (1995). Support-vector networks. Machine Learning.

[25] Breiman L (2001). Random forests. Machine Learning.

[26] Zhang Y, Juhola M (2017). On biometrics with eye movements. IEEE J. Biomed. Health Inf..

[27] Li, X., Joutsijoki, H., Laurikkala, J. & Juhola, M. GDP growth vs. criminal phenomena: data mining of Japan 1926–2013, *Artifcial Intelligence & Society* **33**, 261–274. http://link.springer.com/article/10.1007/s00146-017-0722-7.

[28] Joutsijoki H (2014). Evaluating the performance of artificial neural networks for the classification of freshwater benthic macroinvertebrates. Ecol. Informatics.

[29] Richard, S. *et al*. Standards and guidelines for the interpretation of sequence variants: a joint consensus recommendation of the American College of Medical Genetics and Genomics and the Association for Molecular Pathology. *Genet*. *Med*. **17**, 405-424, 10.1038/gim.2015.30 (2015). 10.1038/gim.2015.30 PMC4544753 25741868

[30] Burke MA, Cook SA, Seidman JG, Seidman SE (2016). Clinical and mechanistic insights into the genetics of cardiomyopathy. Journal of the American College of Cardiology.

[31] Srivastava D, DeWitt N (2016). *In vivo* cellular reprogramming: the next generation. Cell.

[32] Itzhaki I (2011). Calcium handling in human induced pluripotent stem cell derived cardiomyocytes. PLoS ONE.

[33] Karakikes I, Ameen M, Termglinchan V, Wu JC (2015). Human Induced Pluripotent Stem Cell-Derived Cardiomyocytes: Insights into Molecular, Cellular, and Functional Phenotypes. Circ. Res..

[34] Lee YK (2011). Calcium homeostasis in human induced pluripotent stem cell-derived cardiomyocytes. Stem Cell Reviews.

[35] Gherghiceanu M (2011). Cardiomyocytes derived from human embryonic and induced pluripotent stem cells: Comparative ultrastructure. Journal of Cellular and Molecular Medicine.

[36] Lundy SD, Zhu WZ, Regnier M, Laflamme MA (2013). Structural and functional maturation of cardiomyocytes derived from human pluripotent stem cells. Stem Cells and Development.

[37] Laurila, E., Ahola, A., Hyttinen, J. & Aalto-Setälä, K. Methods for *in vitro* functional analysis of iPSC derived cardiomyocytes — Special focus on analyzing the mechanical beating behavior. *Biochimica et Biophysica Acta (BBA) - Molecular Cell Researc*h **1863**(7), Part B, 1864–1872 (2016). 10.1016/j.bbamcr.2015.12.013 26707468

[38] Peters MF, Lamore SD, Guo L, Scott CW, Kolaja KL (2015). Human stem cell-derived cardiomyocytes in cellular impedance assays: bringing cardiotoxicity screening to the front line. Cardiovasc. Toxicol..

[39] Shinnawi R (2015). Monitoring human-induced pluripotent stem cell-derived cardiomyocytes with genetically encoded calcium and voltage fluorescent reporters. Stem Cell Reports.

[40] Herron TJ, Lee P, Jalife J (2012). Optical imaging of voltage and calcium in cardiac cells & tissues. Circ. Res..

[41] Garcia MI, Chen JJ, Boehning D (2017). Genetically encoded calcium indicators for studying long term calcium dynamics during apoptosis. Cell Calcium.

