# Supplementary material for: Detection of genetic cardiac diseases by Ca2+ transient profiles using machine learning methods
Source: Sci Rep. 2018 Jun 19;8:9355. doi: 10.1038/s41598-018-27695-5 (PMC6008430; doi:10.1038/s41598-018-27695-5)
Supplement: Supplementary file 1 — Supplementary Information [file 41598_2018_27695_MOESM1_ESM.docx]

**Detection of genetic cardiac diseases by Ca^2+^ transient profiles using machine learning methods**

Martti Juhola*^1^, Henry Joutsijoki^1^, Kirsi Penttinen^2^ and Katriina Aalto-Setälä^2,3^

^1^Faculty of Natural Sciences, ^2^Faculty of Medicine and Life Sciences, University of Tampere, Finland

^3^Heart Center, Tampere University Hospital, 33520 Tampere, Finland

**Supplementary Note**

**Classification methods used**

In this study, we performed five types of classification approaches:

1. Classification only for the abnormal signals, which included signals from three disease classes (CPVT, HCM and LQT1).
2. Classification only for the normal signals, which included signals from three disease classes (CPVT, HCM and LQT1).
3. Classification for both abnormal and normal signals from three disease classes (CPVT, HCM and LQT1).
4. Classification for normal signals from three disease classes (CPVT, HCM and LQT1) and controls also known as wild types (WT).
5. Classification for both normal and abnormal signals from three disease classes (CPVT, HCM and LQT1) and controls also known as wild types (WT).

The description given below holds for all the above-mentioned cases. We selected a total of nine classification methods to be included in this paper:

1. K-nearest neighbor method (k-NN) ^1,2^
2. Linear discriminant analysis (LDA) ^3,4^
3. Quadratic discriminant analysis (QDA) ^3^
4. Mahalanobis discriminant analysis (MDA) ^5^
5. Decision tree (more specifically CART algorithm) ^6,7^
6. Multinomial logistic regression (MNLR) ^8,9^
7. Naïve Bayes classifier (NB) ^3,4^
8. Random Forest (RF) ^10,11^
9. Binary tree Least-Squares Support Vector Machine (BT-LSSSVM) ^12,13,14^

In addition to these nine classification methods, we tested several variants of k-NN, NB and BT-LSSVM. More specifically, with k-NN we used eight different distance measures:

1. Chebyshev
2. Manhattan
3. Correlation
4. Cosine
5. Euclidean
6. Mahalanobis
7. Standardized Euclidean
8. Spearman

Moreover, three different distance weightings were applied for each distance measure:

1. Equal weighting (weight is 1 for all examples)
2. Inverse weighting (1/dist)
3. Squared inverse weighting (1/dist^2^)

In this way, 24 distance measure and distance weighting combinations were tested with all the three classification approaches described. For NB classifiers, we tested several variations besides the traditional NB classifier. More specifically, we applied the NB classifier with and without kernel density estimation (KDE) ^4^. We performed evaluations with five variants of NB:

1. Traditional NB classifier with Gaussian distribution assumption
2. NB classifier with KDE and Gaussian kernel
3. NB classifier with KDE and box kernel
4. NB classifier with KDE and Epanechnikov kernel
5. NB classifier with KDE and triangle kernel.

Since LSSVM was designed for binary classification problems, it was not directly applicable to our classification task, since we had three disease classes and wild types. Hence, a multi-class extension was needed. There are numerous alternatives for multi-class extensions: we selected a binary tree approach in which one class is eliminated in each layer. Figures 1 and 2 present the constructions used in this study. In the structure, each node corresponds to a binary LSSVM classifier and the numbers 1, 2, 3 and 4 (LQT1=1, HCM=2, CPVT=3, WT=4) indicate classes. The given binary tree structures could be built in a different way, but we decided to use the one we did because the balance between class sizes in a root node was the best one. Since the success of LSSVM is highly dependent on the kernel choice, we chose four kernels for our study:

1. Linear kernel
2. Quadratic kernel
3. Cubic kernel
4. RBF kernel

**3 versus {1,2}**

**3**

**1 versus 2** 2222222221122

**2**

**1**

Figure 1. Binary tree LSSVM multi-class structure used. The numbers 1, 2 and 3 correspond to classes in a dataset. The structure is a peak level structure.

**4 versus {1,2,3}**

**4**

**3 versus {1,2}**

**3**

**1 versus 2**

**2**

**1**

Figure 2. Binary tree LSSVM multi-class structure used. The numbers 1,2,3 and 4 correspond to classes in a dataset. The structure is a peak level structure.

Section 2.1 Classification procedure using parameter-free classification methods

The classification methods LDA, MDA, QDA, MNLR and NB are parameter-free classification methods. We performed classification using the leave-one-signal-out method, in which the data from each signal (in other words, the data derived from each peak within a signal) is once in a test set and N-1 times in a training set in which N is the number of signals in a dataset. A noticeable detail must be remembered with respect to the leave-one-signal-out procedure. Every time we train a classifier, the classifier is trained from the peak-based point of view and not from the signal perspective. A classifier sees each peak as an individual example and not as part of a signal. Hence, when we are predicting a class label for a signal, we first obtain a predicted class label for every peak within a signal; then we must define a predicted class label for a signal based on predicted peak-level class labels. The final class label is determined by evaluating the mode (the most frequent class label) from the predicted peak class labels within a signal. However, this approach constitutes a problem if the mode is not unambiguously determined. Since our dataset includes three disease classes and wild types, we might have the same number of predicted peak class labels from two, three or four classes within a single signal. The general algorithm (used for all classification methods) for solving tie situations is as follows.

When the tie is between two classes:

1. Let the number of classes in a tie be two. Let these classes be C_i_ and C_j_.
2. Collect the training data of classes C_i_ and C_j_ from the training set.
3. Define the number of peaks in each class from the training data obtained in step 2. Let the number of peaks for class C_i_ be P_i_, and for class C_j_ the number of peaks is P_j_. Moreover, we have P_i_+P_j_=P.
4. Generate a random number R from the uniform distribution U(0,1).
5. If R≤P_i_/P, then the final class label for the signal is C_i_. Otherwise, the final class label for the signal is C_j_.

When tie is between three classes:

1. Let the number of classes in a tie be three. Let these classes be C_i_, C_j_ and C_k_.
2. Collect the training data of classes C_i_, C_j_ and C_k_ from the training set.
3. Define the number of peaks in each class from the training data obtained in step 2. Let the number of peaks be P_i_ for class C_i_, P_j_ for class C_j_, and P_k_ for class C_k_. Moreover, we have P_i_+P_j_+P_k_=P.
4. Generate a random number R from the uniform distribution U(0,1).
5. If R≤P_i_/P, then the final class label for the signal is C_i_. If P_i_/P<R≤(P_i_+P_j_)/P, then the final class label for the signal is C_j_. If (P_i_+P_j_)/P<R, the final class label for the signal is C_k_.

When the tie is between four classes:

1. Let the number of classes in a tie be four. Let these classes be C_i_, C_j_, C_k_ and C_m_.
2. Collect the training data of classes C_i_, C_j_, C_k_ and C_m_ from the training set.
3. Define the number of peaks in each class from the training data obtained in step 2. Let the number of peaks be P_i_ for class C_i_, P_j_ for class C_j_, P_k_ for class C_k_, and P_m_ for class C_m_. Moreover, we have P_i_+P_j_+P_k_+P_m_ =P.
4. Generate a random number R from the uniform distribution U(0,1).
5. If R≤P_i_/P, the final class label for the signal is C_i_. If P_i_/P<R≤(P_i_+P_j_)/P, the final class label for the signal is C_j_. If (P_i_+P_j_)/P<R≤ (P_i_+P_j_+P_k_)/P, the final class label for the signal is C_k_. If (P_i_+P_j_+P_k_)/P <R, the final class label for the signal is C_m_.

After having predicted the class label for all signals, we obtained the confusion matrix. From the confusion matrix, we determined the accuracy and true positive rate of the performance measures. Both of these performance measures were computed in percentages, and accuracy was defined as a sum of diagonal elements of a confusion matrix divided by the sum of all elements in a confusion matrix.

Section 2.2 Classification procedure using parameter non-free classification methods

Of the classification methods listed in Section 1, the ones that require parameter values to be specified are DT, k-NN, RF and BT-LSSVM. In all classification methods explained in this subsection, the actual classification procedure was performed using the leave-one-signal-out method in a manner similar to that used for the classification methods given in Section 2.1. The leave-one-signal-out procedure was repeated with all parameter values tested, and the highest accuracy determined the optimal parameter values. Furthermore, possible ties were solved using the same algorithm as presented in Section 2.1. The seed number was used throughout the paper when constructing random numbers from the uniform distribution in order to ensure the reproducibility of results.

For DT we had the following specifications:

1. Each leaf node had a minimum of 1 observation.
2. Each splitting node in the classification tree had at least ten observations.

Besides the selection of distance measure and distance weighting function in a k-NN classifier, an essential issue is the choice of k value. For this study, we selected six k values to be tested: 1, 5, 7, 11, 13 and 17. These values were selected in order to prevent possible ties, which could occur when k value was even or divisible by three (three is the number of disease classes in our dataset). Moreover, if a tie still occurred, it was solved by assigning the test set example to the nearest neighbor class with respect to training set examples.

Random forest (RF) is an ensemble method that has gained popularity. The main parameter for RF is the number of trees. We varied the number of trees in a forest ranging from 1 to 100 with an interval of 1. The best number of trees was decided based on accuracy.

LSSVM is a classification method slightly different from the others: other methods are designed for multi-class classification; LSSVM is not. In multi-class LSSVMs, we must combine the results of many individual binary classifiers. We applied binary tree approaches (Figs. 1 and 2) in modeling our multi-class classification problem. Multi-class LSSVM performance is highly dependent on two factors:

1. Selection of kernel function
2. Selection of parameter values

We described the selected kernels in Section 1. Parameter values are dependent on the kernel chosen. A common parameter value for LSSVM is the regularization parameter, C, which occurs regardless of what kernel is used. The actual hyperparameter of LSSVM occurs in the RBF kernel, which consists of σ (the width of Radial Basis Function). In other words, we must tune up only either parameter C or a parameter combination (C,σ). We decided that the parameter value space is the same for both parameters C and σ. Hence, C, σϵ{2^-12^, 2^-11^, 2^-10^,…, 2^17^}. Thus, we tested 30 different values of C and performed a grid search in which we tested a total of 900 (C,σ) combinations. Since our tree designs include two or three binary LSSVM classifiers, we trained both classifiers with the same parameter values following the guidelines in Hsu and Lin 2002. ^15^ A selection criterion for optimal parameter values was accuracy, and parameter values were tested using the leave-one-signal-out method.

References:

1. Dudani, S.S. The Distance-Weighted k-Nearest-Neighbor Rule. IEEE Transactions on Systems, Man, and Cybernetics **6**(4), 325-327 (1976).

2. Cover, T., Hart, P. Nearest neighbor pattern classification. IEEE Transactions on Information Theory **13**(1), 21-27 (1967).

3. Cios, K.J., Pedrycz, W., Swiniarski, R.W., Kurgan, L.A: Data Mining: A Knowledge Discovery Approach, Springer, New York, NY, USA, 2007.

4. Hastie, T., Tibshirani, R., Friedmann, J. The Elements of Statistical Learning – Data Mining, Inference, and Prediction, Springer, New York, NY, USA, 2^nd^ edition, 2009.

5. Bohling, G. Classical normal-based discriminant analysis, Technical report, Kansas Geological Survey. [http://people.ku.edu/gbohling/EECS833 Accessed 30.6.2016](http://people.ku.edu/gbohling/EECS833%20Accessed%2030.6.2016).

6. Bittencourt, H.R., Clarke, R.T. Use of classification and regression trees (CART) to classify remotely-sensed digital images. Proceedings of the IEEE International Geoscience and Remote Sensing Symposium **6,** 3751-3753 (2003).

7. Duda, R.O., Hart, P.E., Stork, D.G. Pattern Classification, John Wiley & Sons, New York, NY, USA, 2^nd^ edition, 2001.

8. Agresti, A. Categorical Data Analysis, John Wiley & Sons, New York, NY, USA, 1990.

9. Kwak, C., Clayton-Matthews, A. Multinomial logistic regression. *Nursing Research* **51**(6), 404-410 (2002).

10. Breiman, L. Random Forests. *Machine Learning*. **45**(1), 5-32 (2001).

11. Liaw, A., Wiener, M. Classification and regression by random forest. *R News*, **2**(3), 18-22 (2002).

12. Suykens, J.A.K., van Gestel, T., De Brabanter, J., De Moor, B., Vandewalle, J. Least squares support vector machines. World Scientific, New Jersey, USA, 2002.

13. Suykens, J.A.K., Vandewalle, J. Least squares support vector machines. *Neural Processing Letters* **9**(3), 293-300 (1999).

14. Suykens, J.A.K., Vandewalle, J. Multiclass least squares support vector machines. Proceedings of the International Joint Conference on Neural Networks, **2,** 900-903 (1999).

15. Hsu, C.W., Lin, C.J. A comparison of methods for multiclass support vector machines. *IEEE Transactions on Neural Networks* **13**(2), 415-425 (2002).

a
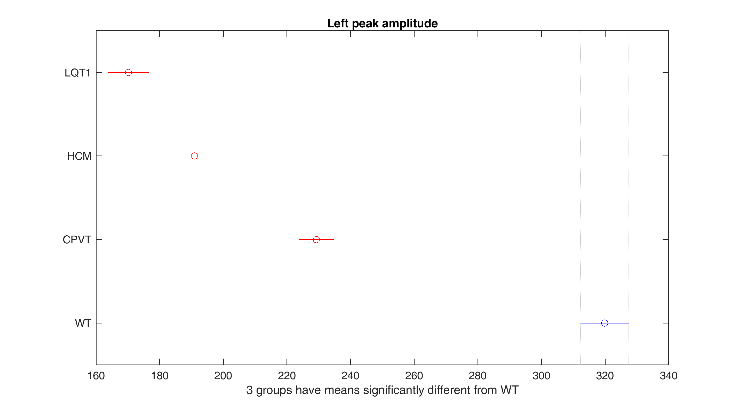
b
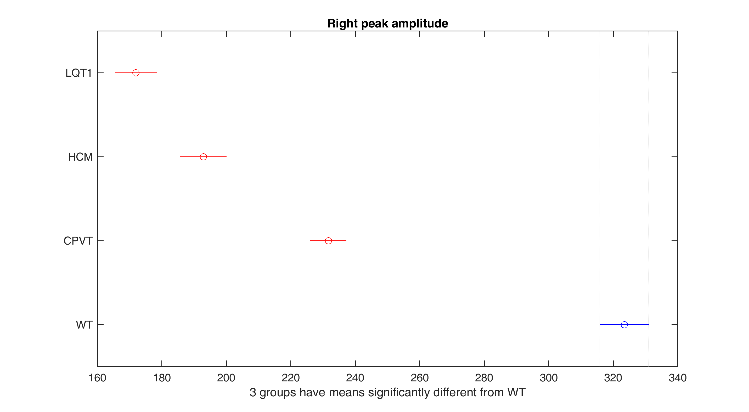


c
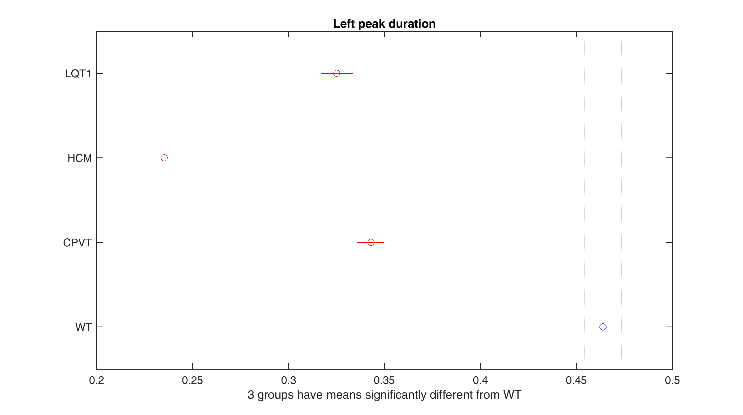
d
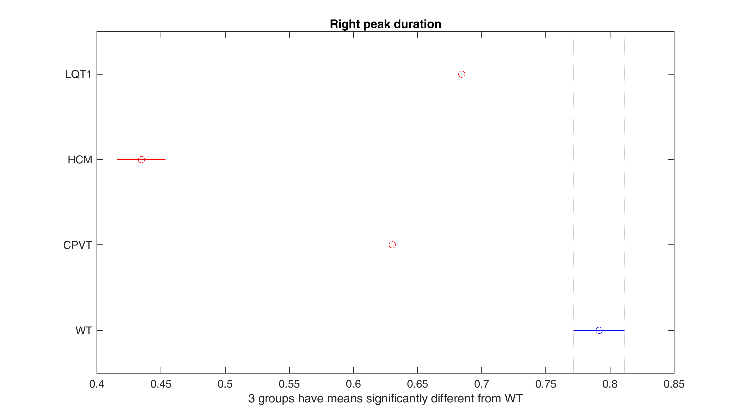


e
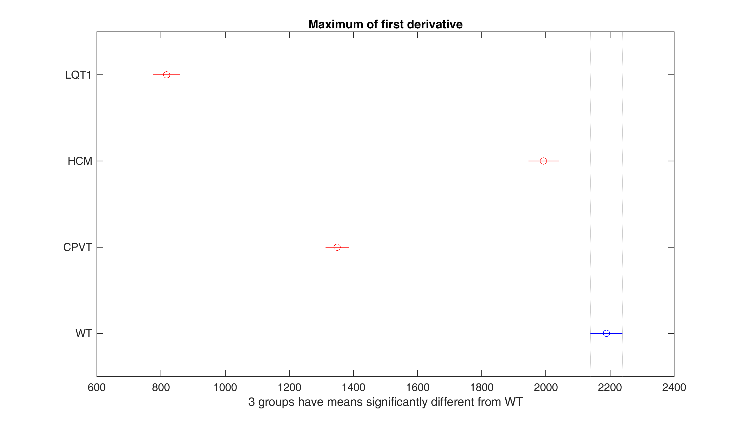
f
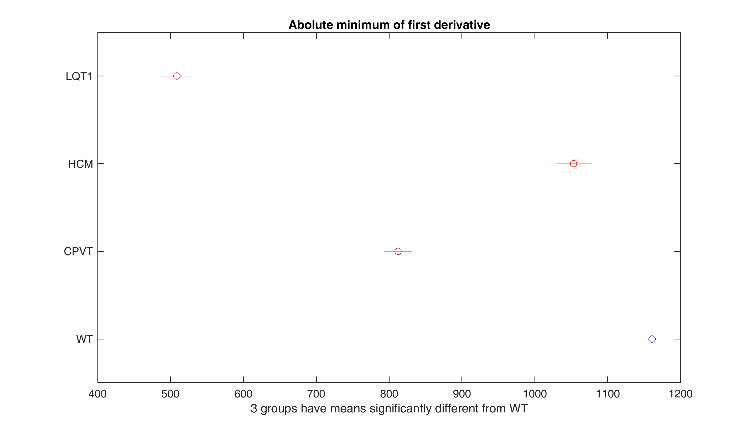


g
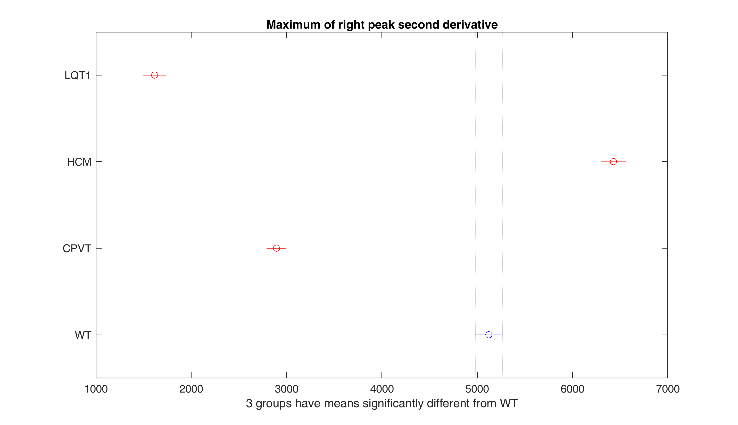
h
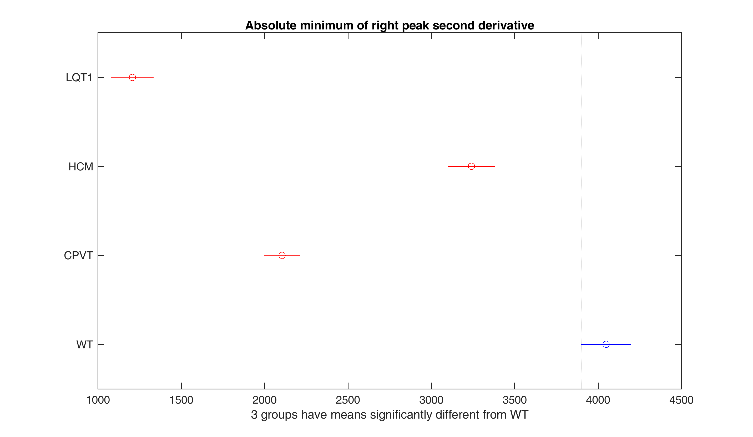


i
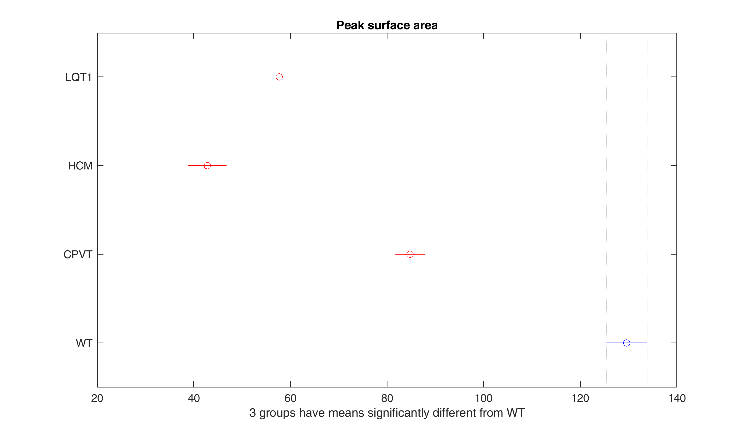
j
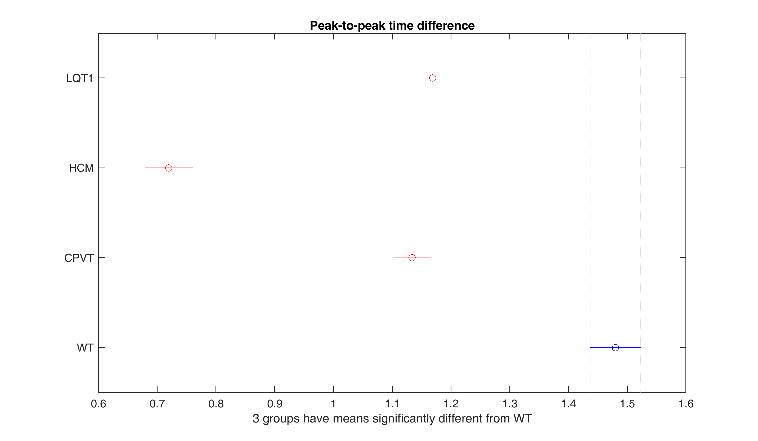


**Supplementary Figure 1a-j.** **Means with 95% confidence limits of ten peak variables.** The vertical axis shows diseases LQT1, HCM, and CPVT and controls (WT). All three diseases differ significantly from the controls in all figures, **a-j**.

**Supplementary Table 1. Cell lines used.**

| Cell line | Disease | Mutation |
| --- | --- | --- |
| UTA.05605.CPVT | CPVT | RyR2* – exon 3 deletion |
| UTA.05208.CPVT | CPVT | RyR2 – P2328S |
| UTA.07001.CPVT | CPVT | RyR2 – T2538R |
| UTA.03701.CPVT | CPVT | RyR2 – L4115F |
| UTA.05503.CPVT | CPVT | RyR2 – Q4201R |
| UTA.05404.CPVT | CPVT | RyR2 – V4653F |
| UTA.00208.LQT1 | LQT1 | KCNQ1* – G589D |
| UTA.00118.LQT1 | LQT1 | KCNQ1 – ivs7-2A>G |
| UTA.07801.HCMM | HCM | MYBPC3*-Gln1061X |
| UTA.06108.HCMM | HCM | MYBPC3-Gln1061X |
| UTA.02912.HCMT | HCM | TPM1*-Asp175Asn |
| UTA.13602.HCMT  UTA.04602.WT | HCM  - | TPM1-Asp175Asn  - |

- Cardiac ryanodine receptor (RyR2), potassium voltage-gated channel subfamily Q member 1 (KCNQ1), myosin-binding protein C (MYBPC3), α-tropomyosin (TPM1).

**Supplementary Table 2. One-way variance analysis results.** Computed *p* values for 60 comparisons of four classes and ten variables: Amplitude of peak left side *A_l_*, amplitude of peak right side *A_r_*, duration of peak left side *D_l_*, duration of peak right side *D_r_*, maximum of the first derivative *s*´ on the left side of a peak, absolute minimum *s*' of the first derivative on the right side of a peak, maximum of the second derivative *s*'' on the right side of a peak, absolute minimum *s*'' of the second derivative on the right side of a peak, area *R* of a peak, and time difference Δ from peak to peak.

|  | Variables | | | | | | | | | |
| --- | --- | --- | --- | --- | --- | --- | --- | --- | --- | --- |
| Comparison | *A_l_* | *A_r_* | *D_l_* [s] | *D_r_* [s] | max *s*' | \|min s'\| | max *s*'' | \|min *s*''\| | *R* | Δ [s] |
| LQT1 vs. HCM | 5.5ˑ10^-4^ | 5.6ˑ10^-4^ | 3.8ˑ10^-9^ | 3.8ˑ10^-9^ | 3.8ˑ10^-9^ | 3.8ˑ10^-9^ | 3.8ˑ10^-9^ | 3.8ˑ10^-9^ | 3.0ˑ10^-6^ | 3.8ˑ10^-9^ |
| LQT1 vs. CPVT | 3.8ˑ10^-9^ | 3.8ˑ10^-9^ | 0.015 | 5.6ˑ10^-5^ | 3.8ˑ10^-9^ | 3.8ˑ10^-9^ | 3.8ˑ10^-9^ | 3.8ˑ10^-9^ | 3.8ˑ10^-9^ | 0.55 |
| LQT1 vs. WT | 3.8ˑ10^-9^ | 3.8ˑ10^-9^ | 3.8ˑ10^-9^ | 3.8ˑ10^-9^ | 3.8ˑ10^-9^ | 3.8ˑ10^-9^ | 3.8ˑ10^-9^ | 3.8ˑ10^-9^ | 3.8ˑ10^-9^ | 3.8ˑ10^-9^ |
| HCM vs. CPVT | 3.8ˑ10^-9^ | 3.8ˑ10^-9^ | 3.8ˑ10^-9^ | 3.8ˑ10^-9^ | 3.8ˑ10^-9^ | 3.8ˑ10^-9^ | 3.8ˑ10^-9^ | 3.8ˑ10^-9^ | 3.8ˑ10^-9^ | 3.8ˑ10^-9^ |
| HCM vs. WT | 3.8ˑ10^-9^ | 3.8ˑ10^-9^ | 3.8ˑ10^-9^ | 3.8ˑ10^-9^ | 1.1ˑ10^-6^ | 2.9ˑ10^-7^ | 3.8ˑ10^-9^ | 3.8ˑ10^-9^ | 3.8ˑ10^-9^ | 3.8ˑ10^-9^ |
| CPVT vs. WT | 3.8ˑ10^-9^ | 3.8ˑ10^-9^ | 3.8ˑ10^-9^ | 3.8ˑ10^-9^ | 3.8ˑ10^-9^ | 3.8ˑ10^-9^ | 3.8ˑ10^-9^ | 3.8ˑ10^-9^ | 3.8ˑ10^-9^ | 3.8ˑ10^-9^ |

**Supplementary Table 3. Separation power values produced by the Scatter method.** For the whole data set, four classes (LQT1, HCM, CPVT and WT) and ten variables. *D_l_*, *D_r_* and Δ are the most important variables and more important than the others.

| Type | Quantities | | | | | | | | | |
| --- | --- | --- | --- | --- | --- | --- | --- | --- | --- | --- |
| Entire data | 0.476 | | | | | | | | | |
| Classes | LQT1 | | | HCM | | | CPVT | | WT | |
|  | 0.513 | | | 0.642 | | | 0.362 | | 0.465 | |
| Variables | *A_l_* | *A_r_* | *D_l_* [s] | *D_r_* [s] | max *s*' | \|min s'\| | Max *s*'' | \|Min *s*''\| | *R* | Δ [s] |
|  | 0.082 | 0.073 | 0.466 | 0.462 | 0.113 | 0.093 | 0.115 | 0.064 | 0.099 | 0.473 |
| Variable ranking | 8 | 9 | 2 | 3 | 5 | 7 | 4 | 10 | 6 | 1 |

**Classification results of three diseases and controls**

**Supplementary Table 4. Normal signals of three diseases: true positive rates (%).** TPRs of LQT1, HCM and CPVT diseases, including 491, 598 and 737 peaks in 28, 37 and 114 signals respectively, and accuracy (%) of all signals (*k*NN is *k* nearest neighbor searching method and LS-SVM least square support vector machine). The best accuracies are bolded.

| Classification method | TPR of LQT1 | TRP of HCM | TRP of CPVT | Accuracy |
| --- | --- | --- | --- | --- |
| *k*NN, Chebychev metric, equal weighting, *k*=1 | 82.1 | 75.7 | 81.6 | 80.4 |
| *k*NN, Chebychev metric, inverse weighting, *k*=5 | 78.6 | 83.8 | 80.7 | 81.0 |
| *k*NN, Chebychev metric, squared inverse weighting, *k*=5 | 82.1 | 86.5 | 82.5 | **83.2** |
| *k*NN, cityblock metric, equal weighting, *k*=1 | 82.1 | 78.4 | 84.2 | 82.7 |
| *k*NN, cityblock metric, inverse weighting, *k*=1 | 82.1 | 78.4 | 84.2 | 82.7 |
| *k*NN, cityblock metric, squared inverse weighting, *k*=1 | 82.1 | 78.4 | 84.2 | 82.7 |
| *k*NN, correlation measure, equal weighting, *k*=7 | 82.1 | 86.5 | 77.2 | 79.9 |
| *k*NN, correlation measure, inverse weighting, *k*=11 | 82.1 | 81.1 | 80.7 | 81.0 |
| *k*NN, correlation measure, squared inverse weighting, *k*=11 | 85.7 | 81.1 | 78.9 | 80.4 |
| *k*NN, cosine measure, equal weighting, *k*=5 | 71.4 | 83.8 | 83.3 | 81.6 |
| *k*NN, cosine measure, inverse weighting, *k*=11 | 75.0 | 83.8 | 83.3 | 82.1 |
| *k*NN, cosine measure, squared inverse weighting, *k*=5 | 71.4 | 83.8 | 84.2 | 82.1 |
| *k*NN, Euclidean metric, equal weighting, *k*=1 | 78.6 | 75.7 | 83.3 | 81.0 |
| *k*NN, Euclidean metric, inverse weighting, *k*=1 | 78.6 | 75.7 | 83.3 | 81.0 |
| *k*NN, Euclidean metric, squared inverse weighting, *k*=1 | 78.6 | 75.7 | 83.3 | 81.0 |
| *k*NN, Mahalanobis metric, equal weighting, *k*=1 | 89.3 | 81.1 | 81.6 | 82.7 |
| *k*NN, Mahalanobis metric, inverse weighting, *k*=1 | 89.3 | 81.1 | 81.6 | 82.7 |
| *k*NN, Mahalanobis metric, squared inverse weighting, *k*=1 | 89.3 | 81.1 | 81.6 | 82.7 |
| *k*NN, standardized Euclidean metric, equal weighting, *k*=1 | 78.6 | 75.7 | 83.3 | 81.0 |
| *k*NN, standardized Euclidean metric, inverse weighting, *k*=1 | 78.6 | 75.7 | 83.3 | 81.0 |
| *k*NN, standardized Euclidean metric, squared inverse weighting, *k*=1 | 78.6 | 75.7 | 83.3 | 81.0 |
| *k*NN, Spearman measure, equal weighting, *k*=1 | 92.9 | 78.4 | 75.4 | 78.8 |
| *k*NN, Spearman measure, inverse weighting, *k*=1 | 92.9 | 78.4 | 75.4 | 78.8 |
| *k*NN, Spearman measure, squared inverse weighting, *k*=1 | 92.9 | 78.4 | 75.4 | 78.8 |
| Linear discriminant analysis | 60.7 | 70.3 | 78.1 | 73.7 |
| Mahalanobis discriminant analysis | 7.1 | 54.1 | 95.6 | 73.2 |
| Quadratic discriminant analysis | 85.7 | 78.4 | 71.9 | 75.4 |
| Decision trees | 71.4 | 81.1 | 84.2 | 81.6 |
| Multinomial logistic regression | 82.1 | 78.4 | 84.2 | 82.7 |
| Naïve Bayes, normal distribution | 75.0 | 70.3 | 59.6 | 64.2 |
| Naïve Bayes, normal kernel | 75.0 | 67.6 | 62.3 | 65.4 |
| Naïve Bayes, box kernel | 71.4 | 67.6 | 63.2 | 65.4 |
| Naïve Bayes, Epanechnikov kernel | 71.4 | 67.6 | 63.2 | 65.4 |
| Naïve Bayes, triangle kernel | 75.0 | 67.6 | 63.2 | 65.9 |
| Random forests, 65 trees | 75.0 | 81.1 | 90.4 | **86.0** |
| LS-SVM linear kernel, parameter *C*=2^9^ | 35.7 | 73.0 | 79.8 | 71.5 |
| LS-SVM quadratic kernel, parameter *C*=2^-7^ | 60.7 | 64.9 | 86.8 | 78.2 |
| LS-SVM cubic kernel, parameter *C*=2^5^ | 75.0 | 75.7 | 85.1 | 81.6 |
| LS-SVM RBF kernel, parameters *C*=2^3^, sigma=2 | 78.6 | 78.4 | 87.7 | **84.4** |

**Supplementary Table 5. Abnormal signals of three diseases: true positive rates (%)**. TPRs of LQT1, HCM and CPVT diseases with 1144, 746 and 1574 peaks in 62, 34 and 119 signals respectively, and accuracy (%) of all signals (*k*NN is *k* nearest neighbor searching method and LS-SVM least square support vector machine). The best accuracies are bolded.

| Classification method | TPR of LQT1 | TPR of HCM | TPR of CPVT | Accuracy |
| --- | --- | --- | --- | --- |
| *k*NN, Chebychev metric, equal weighting, *k*=1 | 82.3 | 85.3 | 79.0 | 80.9 |
| *k*NN, Chebychev metric, inverse weighting, *k*=1 | 82.3 | 85.3 | 79.0 | 80.9 |
| *k*NN, Chebychev metric, squared inverse weighting, *k*=1 | 82.3 | 85.3 | 79.0 | 80.9 |
| *k*NN, cityblock metric, equal weighting, *k*=1 | 83.9 | 88.2 | 77.3 | 80.9 |
| *k*NN, cityblock metric, inverse weighting, *k*=1 | 83.9 | 88.2 | 77.3 | 80.9 |
| *k*NN, cityblock metric, squared inverse weighting, *k*=7 | 90.3 | 79.4 | 78.2 | 81.9 |
| *k*NN, correlation measure, equal weighting, *k*=11 | 91.9 | 79.4 | 73.1 | 79.5 |
| *k*NN, correlation measure, inverse weighting, *k*=5 | 85.5 | 79.4 | 76.5 | 79.5 |
| *k*NN, correlation measure, squared inverse weighting, *k*=1 | 85.5 | 82.4 | 74.8 | 79.1 |
| *k*NN, cosine measure, equal weighting, *k*=1 | 87.1 | 85.3 | 78.2 | 81.9 |
| *k*NN, cosine measure, inverse weighting, *k*=1 | 87.1 | 85.3 | 78.2 | 81.9 |
| *k*NN, cosine measure, squared inverse weighting, *k*=1 | 87.1 | 85.3 | 78.2 | 81.9 |
| *k*NN, Euclidean metric, equal weighting, *k*=1 | 83.9 | 85.3 | 79.0 | 81.4 |
| *k*NN, Euclidean metric, inverse weighting, *k*=1 | 83.9 | 85.3 | 79.0 | 81.4 |
| *k*NN, Euclidean metric, squared inverse weighting, *k*=1 | 83.9 | 85.3 | 79.0 | 81.4 |
| *k*NN, Mahalanobis metric, equal weighting, *k*=1 | 91.9 | 85.3 | 81.5 | **85.1** |
| *k*NN, Mahalanobis metric, inverse weighting, *k*=1 | 91.9 | 85.3 | 81.5 | **85.1** |
| *k*NN, Mahalanobis metric, squared inverse weighting, *k*=1 | 91.9 | 85.3 | 81.5 | **85.1** |
| *k*NN, standardized Euclidean metric, equal weighting, *k*=1 | 83.9 | 85.3 | 79.0 | 81.4 |
| *k*NN, standardized Euclidean metric, inverse weighting, *k*=1 | 83.9 | 85.3 | 79.0 | 81.4 |
| *k*NN, standardized Euclidean metric, squared inverse weighting, *k*=1 | 83.9 | 85.3 | 79.0 | 81.4 |
| *k*NN, Spearman measure, equal weighting, *k*=13 | 90.3 | 76.5 | 73.1 | 78.6 |
| *k*NN, Spearman measure, inverse weighting, *k*=7 | 83.9 | 79.4 | 78.2 | 80.0 |
| *k*NN, Spearman measure, squared inverse weighting, *k*=11 | 83.9 | 79.4 | 79.8 | 80.9 |
| Linear discriminant analysis | 59.7 | 61.8 | 81.5 | 72.1 |
| Mahalanobis discriminant analysis | 46.8 | 61.8 | 97.5 | 77.2 |
| Quadratic discriminant analysis | 90.3 | 85.3 | 52.1 | 68.4 |
| Decision trees | 67.7 | 82.4 | 84.0 | 79.1 |
| Multinomial logistic regression | 72.6 | 76.5 | 77.3 | 75.8 |
| Naïve Bayes, normal distribution | 87.1 | 67.6 | 28.6 | 51.6 |
| Naïve Bayes, normal kernel | 72.6 | 73.5 | 48.7 | 59.5 |
| Naïve Bayes, box kernel | 77.4 | 61.8 | 47.1 | 58.1 |
| Naïve Bayes, Epanechnikov kernel | 72.6 | 64.7 | 48.7 | 58.1 |
| Naïve Bayes, triangle kernel | 72.6 | 70.6 | 49.6 | 59.5 |
| Random forests, 37 trees | 85.5 | 85.3 | 86.6 | **86.0** |
| LS-SVM linear kernel, parameter *C*=2^2^ | 61.3 | 88.2 | 73.1 | 72.1 |
| LS-SVM quadratic kernel, parameter *C*=2^3^ | 77.4 | 82.4 | 79.8 | 79.5 |
| LS-SVM cubic kernel, parameter *C*=2^-4^ | 90.3 | 76.5 | 84.0 | 84.7 |
| LS-SVM RBF kernel, parameters *C*=2^2^, sigma=1 | 85.5 | 79.4 | 86.6 | **85.1** |

**Supplementary Table 6. Normal signals of three diseases and controls: true positive rates (%).** TPRs of LQT1, HCM, CPVT diseases and controls (WT) with 491, 598, 737 and 1023 peaks in 28, 37, 114 and 120 signals respectively, and accuracy (%) of all signals (*k*NN is *k* nearest neighbor searching method and LS-SVM least square support vector machine). Five best results are only presented. The best accuracies are bolded.

| Classification method | TPR of LQT1 | TPR of HCM | TPR of CPVT | TPR of WT | Accuracy |
| --- | --- | --- | --- | --- | --- |
| *k*NN, Mahalanobis metric, equal weighting, *k*=1 | 89.3 | 73.0 | 64.9 | 85.0 | **76.3** |
| *k*NN, Mahalanobis metric, inverse weighting, *k*=1 | 89.3 | 73.0 | 64.9 | 85.0 | **76.3** |
| *k*NN, Mahalanobis metric, squared inverse weighting, *k*=1 | 89.3 | 73.0 | 64.9 | 85.0 | **76.3** |
| Random forests, 68 trees | 78.6 | 59.5 | 63.2 | 85.8 | 73.2 |
| LS-SVM RBF kernel, parameters *C*=2^2^, sigma=2 | 60.7 | 70.3 | 70.2 | 82.5 | 74.2 |
